# Aqueous humor concentrations of vascular endothelial growth factor and pigment epithelium-derived factor in high myopic patients

**Published:** 2012-08-14

**Authors:** Young Joo Shin, Woo Ho Nam, Soo Eun Park, Joo Hyun Kim, Ha Kyoung Kim

**Affiliations:** Department of Ophthalmology, Hallym University College of Medicine, Seoul, Korea

## Abstract

**Purpose:**

To compare the aqueous humor levels of vascular endothelial growth factor (VEGF) and pigment epithelium-derived factor (PEDF) in high myopic eyes and control eyes.

**Methods:**

Aqueous humor samples were collected from 21 highly myopic eyes of 20 patients (high myopia group) and from 30 cataract eyes of 30 patients with no choroidal neovascularization (CNV) or other ocular or systemic diseases (control group). Of the 21 high myopic eyes, 13 had no complications secondary to high myopia (high myopia with no complications group), 3 had posterior staphyloma (high myopia with staphyloma group), and 5 had chorioretinal atrophy (high myopia with chorioretinal atrophy group). The aqueous humor levels of VEGF and PEDF were determined by using commercially available enzyme-linked immunosorbent assay kits.

**Results:**

Aqueous humor levels of VEGF were significantly lower in the high myopia group compared to that in the control group (p<0.001). VEGF levels decreased with an increase in the axial length (p<0.001). PEDF levels tended to be higher in the high myopia group compared to that in the control group; however, the difference was not significant. Three high myopia groups had significantly lower VEGF/PEDF ratios than the control group (p=0.000, 0.002, and 0.005).

**Conclusions:**

Aqueous humor levels of VEGF in the high myopia group were significantly lower than those in the control group. The differing levels of VEGF and PEDF in the high myopia and control groups suggest that high myopia disrupts the VEGF/PEDF balance in retinal pigment epithelium (RPE) cells.

## Introduction

High myopia is associated with degenerative changes such as thinning of the retinal pigment epithelium, chorioretinal atrophy, posterior staphyloma, lattice degeneration, and choroidal neovascularization (CNV) in the posterior segment of the eye [1-3]. Conversely, diabetic retinopathy is less severe in myopic patients, and myopic refraction and a longer axial length are associated with a lower risk of diabetic retinopathy, particularly vision-threatening retinopathy [4-6].

Vascular endothelial growth factor (VEGF) is an endothelial cell mitogen and a vasopermeability factor [7]. VEGF plays an essential role in ischemic retinal neovascularization and CNV secondary to age-related macular degeneration [8-12]. In contrast, pigment epithelium-derived factor (PEDF) acts as an anti-angiogenesis [13], an anti-inflammatory [14,15], or a neuroprotective factor [16]. There has been several studies about the role of VEDF and PEDF in development of CNV, and anti-VEGF therapy has been used for treating CNV. In the previous study [17,18], it has been reported that the VEGF concentration in the aqueous humor of patients with myopic CNV is lower than in normal controls [17] and there are significantly lower concentrations of VEGF in myopic eyes than in hyperopic eyes [18]. However, it is unknown whether these results are due to dilution effect in larger eyes or degeneration of retinal pigment epithelium (RPE) and choroid.

In this study, we classified the patients according to the severity of RPE degeneration and compared the aqueous levels of VEGF and PEDF in highly myopic and control eyes.

## Methods

This comparative control study investigated the aqueous humor levels of VEGF and PEDF in highly myopic eyes. For controls, aqueous humor samples were collected from senile cataract patients free from other ocular or systemic diseases. The study protocol complied with the provisions of the Declaration of Helsinki and was reviewed and approved by the Institutional Review Board/Ethics Committee of Hallym University Medical Center, Seoul, Korea. Patients were enrolled from the Ophthalmic Centers in Hallym University Kangnam Sacred Heart hospital from July to December, 2010.

All patients underwent a complete ophthalmic examination, including refraction, measurements of the axial length and best-corrected visual acuity, indirect stereoscopic ophthalmoscopy, fluorescein angiography, and color fundus photography. The high myopic eyes were divided into three groups; high myopia with no complications group, high myopia with posterior staphyloma group, and high myopia with chorioretinal atrophy group. The high myopia with no complications group was defined as a group without degenerative complications including chorioretinal atrophy and posterior staphyloma; chorioretinal atrophy group was defined as thinning of the retinal pigment epithelium and choroid with resulting atrophic appearance of the fundus; and the posterior staphyloma group was diagnosed when the ectasia was visualized.

### Sample collection

Undiluted aqueous humor samples were collected from patients with high myopic eyes and from the senile cataract patients (control group). In the high myopic eyes and the cataract patients, before cataract surgery, anterior chamber paracentesis was performed and no steroids were administered. Aqueous humor samples were collected in sterile tubes and stored at −80 °C until analysis.

### Measurement of VEGF and PEDF by using ELISA

The aqueous humor levels of VEGF and PEDF were measured using the commercially available VEGF Quantikine enzyme-linked immunosorbent assay (ELISA) kit (R&D systems, Minneapolis, MN) and PEDF sandwich ELISA kit (Chemicon International, Temecula, CA), respectively, according to the respective manufacturer’s instructions. Briefly, for the VEGF assay, aqueous humor samples were added to each well of a microtiter plate that was precoated with anti-mouse VEGF polyclonal antibody and incubated for 2 h. Then, each well was washed with a wash buffer and incubated for an additional 2 h with 100 μl of enzyme-linked polyclonal antibody specific for mouse VEGF. After further washing, a substrate solution was added to each well. The plate was incubated for 30 min at room temperature, following which, the enzyme reaction was stopped, and the color intensity of the reaction mixture was measured at 450 nm by using a multi-plate reader (Lambda Bio-20; Beckman Coulter, Inc., Brea, CA). For the PEDF assay, aqueous humor samples were first incubated for 1 h with 8 mol/l of urea (Sigma-Aldrich, Inc., St. Louis, MO) and then were diluted 1:200. The diluted solution was then applied to the microtiter plate. After incubation at 37 °C for 1 h and extensive washing, this plate was incubated for 1 h with 100 μl of a biotinylated mouse anti-PEDF antibody, followed by incubation for 1 h with 100 μl of streptavidin-peroxidase conjugate. Then, 3,3′,5,5′-tetramethylbenzidine (TMB/E) was added to each well and incubated for 5–10 min, following which, the plate was read at 450 nm by the multi-plate reader (Lamboda Bio-20). Serial dilutions of recombinant human VEGF and PEDF served as standards.

### Statistics

Experimental data were expressed as mean±SD. The results were analyzed using the Mann–Whitney test, *t*-test, and Pearson correlation test. All statistical analyses were performed using SPSS version 14.0 (SPSS inc., Chicago, IL). A p value of <0.05 was considered significant in all cases.

## Results

Aqueous humor samples were collected from 21 highly myopic eyes of 20 patients (high myopia group) and 30 cataract eyes of 30 patients with no choroidal neovascularization (CNV) or other ocular or systemic diseases (control group). Of the 21 highly myopic eyes, 13 had no complications (high myopia with no complications group), 3 had posterior staphyloma (high myopia with posterior staphyloma group), and 5 had chorioretinal atrophy (high myopia with chorioretinal atrophy group). The mean age of the patients was 65.3±11.5 years. Of the 51 eyes, 36 belonged to female patients and 15 to male patients. The high myopia and control groups had a mean refractive error of −11.80±3.60 and 0.83±1.71 D, respectively, and a mean axial length of 27.97±2.27 and 23.03±0.61 mm, respectively (Table 1).

**Table 1:** Characteristics of subjects.

**Varient**	**Normal control**	**High myopia without complications**	**High myopia with chorioretinopathy**	**High myopia with post. staphyloma**
Age (yr)	67.6±10.8	61.0±9.6	66.40±19.13	77.3±0.58
Refractive error (D)	0.83±1.71	-11.62±3.44	-12.40±4.76	-10.8±1.39
Axial length (mm)	23.03±0.61	27.97±2.27	28.48±1.22	29.57±0.71

### VEGF levels

**Figure 1:**
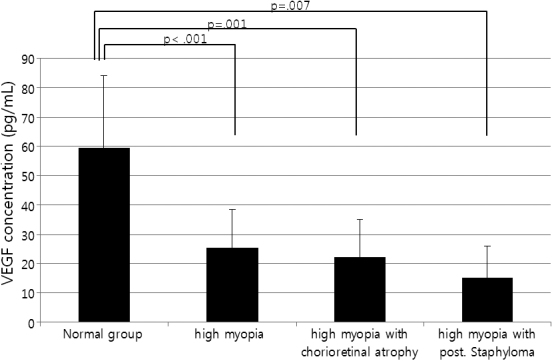
Aqueous humor VEGF levels. The 3 high myopia groups had lower VEGF concentrations compared to the control group (p<0.001, p=0.001, and p=0.007; Mann–Whitney test). The high myopia with posterior staphyloma group had the lowest VEGF levels.

The aqueous humor levels of VEGF in the high myopia and control groups were 26.12±13.74 and 59.46±24.64 pg/ml, respectively. VEGF levels in the high myopia group were significantly lower compared to that in the control group (p<0.01, *t*-test). All 3 high myopia groups had lower VEGF levels compared to the control group (p<0.001, p=0.001, and p=0.007, respectively; Mann–Whitney test). The high myopia with posterior staphyloma group reported the lowest VEGF levels (Figure 1, Table 2).

**Table 2:** VEGF and PEDF concentrations and VEGF/PEDF ratio. Experimental data were expressed as mean±SD.

**Group**	**VEGF concentration (pg/ml)**	**PEDF concentration (ng/ml)**	**VEGF/PEDF ratio (pg/ng)**
Normal control	59.46±24.64	3.08±1.89	24.49±16.12
High myopia without complications	26.12±13.74	4.11±2.31	8.78±7.28
High myopia with chorioretinopathy	22.15±13.04	3.90±3.01	6.52±2.64
High myopia with post. staphyloma	15.38±10.52	6.21±1.75	2.88±2.16

### PEDF levels

**Figure 2:**
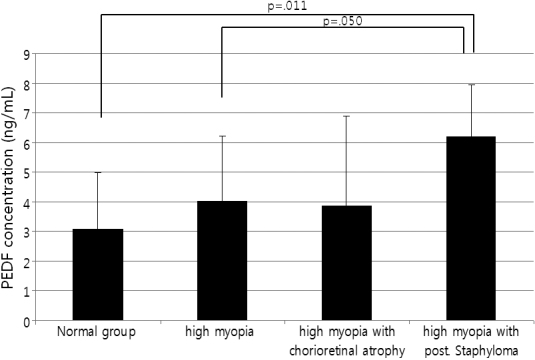
Aqueous humor PEDF levels. The high myopia with no complications group and high myopia with posterior staphyloma group had higher PEDF levels compared to the control group and high myopia with no complications group (p=0.011 and 0.050, respectively; Mann–Whitney test).

The aqueous humor levels of PEDF in the high myopia and control groups were 4.01±2.21 and 3.08±1.89 pg/ml, respectively. PEDF concentrations did not differ significantly between the high myopia and control groups (p=0.086; *t*-test). The high myopia with posterior staphyloma group had higher PEDF concentrations than the control group or high myopia with no complications group (p=0.011 and 0.050, respectively; Mann–Whitney test; Figure 2, Table 2).

### VEGF/PEDF ratios

**Figure 3:**
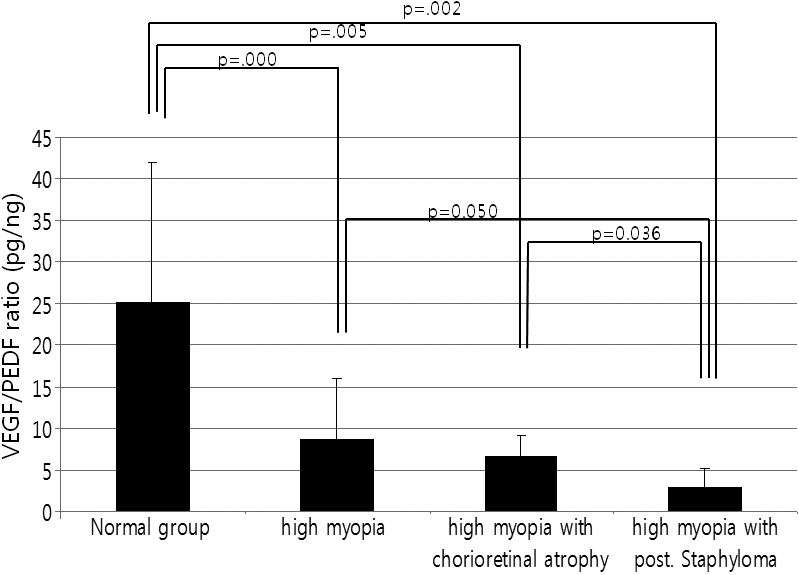
VEGF/PEDF ratio in the control group and in high myopic patients. The 3 high myopia groups had a significantly lower VEGF/PEDF ratio than the control group (p=0.000, 0.005, and 0.002; Mann–Whitney test). In particular, high myopic patients with posterior staphyloma had a significantly lower VEGF/PEDF ratio than the high myopia with no complications group and high myopia with chorioretinal atrophy group (p=0.050 and 0.036, respectively; Mann–Whitney test).

The 3 high myopia groups had significantly lower VEGF/PEDF ratios than the control group (p=0.000, 0.002, and 0.005; Mann–Whitney test). The high myopia with posterior staphyloma group had a significantly lower VEGF/PEDF ratio than the high myopia with no complications group and high myopia with chorioretinal atrophy group (p=0.050 and 0.036, respectively; Mann–Whitney test). However, the VEGF/PEDF ratios in the high myopia with chorioretinal atrophy group did not differ from those in the high myopia with no complications group (Figure 3, Table 2).

### Correlation between axial length and the levels of VEGF and PEDF

**Figure 4:**
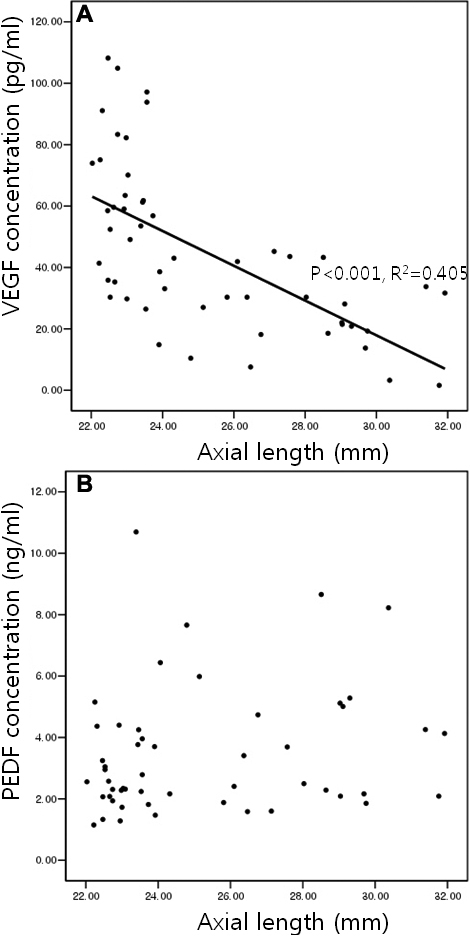
Correlation between axial length and aqueous humor levels of VEGF and PEDF. VEGF concentrations depend on the axial length of the eyeball (p<0.001; r2=0.405; Pearson correlation test; **A**) while PEDF concentrations are not significant (p=0.169; Pearson correlation test; **B**).

VEGF levels decreased with an increase in the axial length of the eyeball (p<0.001; r^2^=0.405; Pearson correlation test), while no significant correlation was found between the axial length and the PEDF levels (p=0.169, Pearson correlation test; Figure 4A,B).

## Discussion

High myopia is a degenerative disease [19] that is associated with a low risk of ischemic retinal diseases including diabetic retinopathy [5]. VEGF is an angiogenic and a vasopermeable factor [20,21]. PEDF is an intrinsic anti-angiogenic factor [13] and an anti-inflammatory factor [14,15]. This factor regulates angiogenesis and cell proliferation via a negative feedback mechanism [22]. VEGF is secreted by the retinal pigment epithelium (RPE) cells [20,21], while PEDF is synthesized and secreted by the RPE as well as the retinal ganglion cells and diffuses into the vitreous and the aqueous humors [23]. A balance between the levels of VEGF and PEDF in the retina is important. A high VEGF and low PEDF level is associated with vision-threatening diseases, including proliferative diabetic retinopathy and CNV [8-12]. Different from the previous study [17,18], we classified the patients according to the severity of retinal degeneration and compared the aqueous levels of VEGF and PEDF in normal eyes and high myopia without CNV.

In this study, the aqueous humor levels of VEGF in the high myopia group was lower compared to that in the control group; indeed the group with more severe degenerative changes had the lowest aqueous humor levels of VEGF. VEGF is secreted by differentiated RPE cells [22]. In high myopia, RPE cells exhibit generalized degenerative changes, and loss of function in RPE cells leads to photoreceptor degeneration [24]. Thus, VEGF production may decrease in high myopia due to degenerative RPE and choriocapillaries [25]. The PEDF concentrations in the high myopic group did not differ significantly from that in the control group. However, these levels were markedly higher in the high myopia with posterior staphyloma group compared to that in the control group (p=0.011) and in the high myopia with no complications group (p=0.050). Atrophy of the RPE and choriocapillaries has been reported within the staphyloma [19,26]. Because VEGF down-regulates PEDF [12], PEDF overproduction may be attributed to a lack of counteracting VEGF. VEGF and PEDF play a role by a regulatory interaction between the counterbalancing angiogenic actions of stimulators and inhibitors [20]. PEDF is moderately antagonistic toward neovascularization development [20]. The VEGF/PEDF ratio has been described to suggest the ability of the eyes to counterbalance the effects of VEGF by producing PEDF [20]. In this study, VEGF/PEDF ratios were significantly lower in all 3 high myopia groups compared to that in the control group (p=0.000, 0.002, and 0.005; Mann–Whitney test). These findings suggest that VEGF secretion may be more influenced by RPE degeneration than by PEDF secretion and that the VEGF/PEDF balance may be disrupted in degenerative RPE cells. In particular, the high myopia with posterior staphyloma group had significantly lower VEGF/PEDF ratios than the high myopia with no complications group and high myopia with chorioretinal atrophy group (p=0.050 and 0.036, respectively; Mann–Whitney test) although axial lengths and refractive errors between high myopia groups are a little different (Table 1). These results suggest that high myopia with posterior staphyloma disrupts the VEGF/PEDF balance in RPE cells.

This study has several limitations that need to be taken into account. The possible limitation of this study is the relatively small sample size. However, this study is the first study to show that the VEGF/PEDF ratios in high myopia are low and explain why high myopia is associated with low risk of diabetic retinopathy.

In conclusion, we found that the aqueous humor concentrations of VEGF in high myopia group were significantly lower compared to that in the controls group. The differing concentrations of both VEGF and PEDF in the high myopia and control groups suggest that high myopia disrupts the VEGF/PEDF balance in RPE cells. Further studies are warranted to enhance our understanding of high myopia.
